# Distinct Illness Representation Profiles Are Associated With Anxiety in Women Testing Positive for Human Papillomavirus

**DOI:** 10.1093/abm/kaab022

**Published:** 2021-04-21

**Authors:** Emily McBride, Laura A V Marlow, Joseph Chilcot, Rona Moss-Morris, Jo Waller

**Affiliations:** 1 Department of Behavioural Science and Health, Institute of Epidemiology and Health Care, University College London, London, UK; 2 Cancer Prevention Group, School of Cancer and Pharmaceutical Sciences, King’s College London, London, UK; 3 Department of Psychology, Institute of Psychiatry, Psychology and Neuroscience, King’s College London, London, UK

**Keywords:** Human papillomavirus, HPV, Illness perception, Illness representation, Latent profile analysis, Anxiety, Cancer, Cancer screening

## Abstract

**Background:**

Testing positive for human papillomavirus (HPV) at cervical cancer screening has been associated with heightened anxiety. To date, the cognitive determinants of heightened anxiety remain unclear, making it difficult to design effective interventions.

**Purpose:**

This study investigated latent illness representation profiles in women testing positive for HPV with no abnormal cells (normal cytology) and explored associations between these profiles and anxiety.

**Methods:**

Women aged 24–66 (*n* = 646) who had tested HPV-positive with normal cytology at routine HPV primary screening in England completed a cross-sectional survey shortly after receiving their result.

**Results:**

Latent profile analysis identified three distinct profiles of illness representations (termed “adaptive,” “negative,” and “negative somatic”), which differed significantly in their patterns of illness perceptions. Hierarchal linear regression revealed that these latent illness representation profiles accounted for 21.8% of the variance in anxiety, after adjusting for demographic and clinical characteristics. When compared with adaptive representations (Profile 1), women with negative representations (Profile 2) and negative somatic representations (Profile 3) had significantly higher anxiety, with clinically meaningful between-group differences (mean difference [MD] = 17.26, confidence interval [CI]: 14.29–20.22 and MD = 13.20, CI: 9.45–16.96 on the S-STAI-6, respectively).

**Conclusion:**

The latent illness representation profiles identified in this study provide support for the role of negative beliefs contributing to anxiety in women testing HPV-positive with normal cytology. Characteristics specific to subgroups of highly anxious women (Profiles 2 and 3) could be used by policymakers to target information in routine patient communications (e.g., test result letters) to reduce unnecessary burden. Future research should adopt longitudinal designs to understand the trajectory of illness representations from HPV diagnosis through to clearance versus persistence.

## Teaser

Women testing HPV-positive at cervical screening displayed three different sets of beliefs about HPV (three profiles). These profiles helped to explain their anxiety levels.

## Introduction

Human papillomavirus (HPV) is a common sexually transmitted infection responsible for over 99% of cervical cancers. In England, HPV primary cervical screening was fully implemented in 2020, and around 270,000 women (8.5% of those attending screening) are now expected to test HPV-positive with normal cytology (normal cervical cells; HPV+/normal) each year [1]. Due to the absence of cervical cell abnormalities, an HPV+/normal result carries a very low absolute risk of cervical cancer; however, given that HPV has been detected, relative risk is higher than average and women are recalled for repeat screening at 12 months rather than the standard 3 or 5 year recall, depending on age. Most HPV infections clear naturally within 18 months (65%) [2]. Therefore, women are only referred to colposcopy, which is a follow-up diagnostic procedure to examine the cervix, if they test HPV+/normal three consecutive times at 12 month intervals [1].

The psychological evaluation of HPV primary screening in England found that women testing HPV+/normal displayed higher short-term anxiety than those with normal screening results, as well as elevated psychosexual distress for up to 12 months [3, 4]. Despite low cancer risk, nearly a quarter experienced clinically significant anxiety [4]. A recent mixed methods systematic review identified cognitive constructs associated with adverse emotional response in women testing HPV-positive, such as concerns about cancer and relationship infidelity. However, most studies included women with HPV accompanied by abnormal cytology, where cancer risk is greater than with an HPV+/normal result [5]. To date, there has been little psychological research in women testing HPV+/normal at routine HPV primary screening and no explanatory quantitative research exploring associations between psychological outcomes and anxiety.

Leventhal’s Common-Sense Model of Self-Regulation (CSM) aims to explain variations in adaption to illness and health outcomes based on a dual model of cognitive and affective processes [6–8]. Illness representations, which form part of the cognitive pathway of the CSM, refer to sets of beliefs and expectations about an illness or somatic symptom. Illness representations combine five illness perception dimensions: (i) identity (perceived symptoms related to condition); (ii) timeline (perceived onset, duration, and decline); (iii) consequences (experienced and anticipated physical, cognitive, and social disruption); (iv) control (perceived personal and treatment control); and (v) cause (perceived cause of condition) [7]. Illness coherence (perceived understanding of condition) and emotion (negative emotion due to condition) were later conceptualized as additional illness perception dimensions [9]. To date, most studies focusing on illness representations across health conditions have treated each illness perception dimension as a separate variable. They have assessed the influence of individual perceptions on health outcomes, often in multivariate analyses that include all or some of the dimensions. Five meta‐analyses of studies measuring individual illness perceptions found a lack of consistency in the way that each of the dimensions related to psychological and behavioral outcomes [10–14]. Concerns have been raised about the lack of reproducibility of findings for the CSM [15].

Given that illness perceptions are thought to form overarching representations, treating them as separate perceptions oversimplifies the intended pathways within the CSM [7, 8, 16]. In part, the examination of individual illness perceptions in previous studies may explain inconsistencies and heterogeneity in findings. Some recent studies have adopted analytic clustering approaches to identify subgroups within a sample with shared patterns of illness perceptions [17–20]. Unlike the traditional approach of examining individual illness perceptions, clustering approaches make it possible to identify distinct groups of illness perception profiles more typical to the concept of overarching illness representations [21, 22]. These latent illness representations can be examined to assess their relation to health outcomes and psychological adjustment. An advantage of adopting a clustering approach is that interventions can be tailored to specific high-risk groups within a population, targeting sets of beliefs known to contribute to adverse outcomes [23]. A systematic review explored studies adopting analytic clustering approaches for illness representations in chronic health conditions and identified only 12 studies across conditions such as hypertension, diabetes, and breast cancer [24]. The illness representation clusters associated with favorable health-related outcomes included perceptions of lower consequences, fewer symptoms, lower negative emotion, and more stable disease patterns. No studies were identified adopting this approach in HPV, cervical cancer, or cervical screening populations; therefore, it is unknown whether the same patterns would be observed.

Despite a body of psychological research in HPV and cervical screening, there have been no studies examining individual illness perceptions or overarching illness representations associated with testing HPV-positive. The primary objective of this study was to investigate whether women testing HPV+/normal had distinct patterns of illness perceptions in order to identify illness representation profiles. The secondary objective was to determine the extent to which each illness representation profile is associated with anxiety after adjusting for relevant demographic and clinical characteristics. We hypothesized that there would be two or more distinct latent illness representation profiles, which would each display different associations with anxiety.

## Method

### Participants and Design

Women aged 24–66 who had tested HPV+/normal for the first time, or second or third consecutive time, were recruited to a cross-sectional survey study through two National Health Service Cervical Screening Programme (NHSCSP) HPV primary screening sites in England (North West London and Central Manchester). They were mailed a survey within 3 weeks of receiving their result. Health Research Authority (HRA) approval was granted on January 9, 2019 (Research Ethics Committee reference: 18/EM/0227 and Confidentiality Advisory Group reference: 18/CAG/0118). Cervical Screening Research Advisory Committee approval was granted on March 15, 2019 (ODR1819_005). This study was registered (ISRCTN15113095) with recruitment between April 5, 2019 and April 21, 2020.

### Study Procedures

Potential participants were identified by National Health Service (NHS) staff at two clinical sites. Women were mailed a study invitation letter, information sheet, survey, and prepaid return envelope. A reminder pack containing the same information was mailed 3 weeks later. Consent was implied through the completion of a survey. NHS laboratories provided information on all approached participants for age, index of multiple deprivation score, test result, and anticipated date of test result delivery.

### Measures

The primary study outcome (dependent) measure was state anxiety (S-STAI-6) [25]). Predictor (independent) variables included eight illness perceptions (Brief Illness Perception Questionnaire [BIPQ]) [26], which were subject to latent profile analysis (LPA) in order to identify illness representation profiles. Covariates included demographic and clinical variables.

### Anxiety (S-STAI-6)

The short‐form state anxiety inventory (S‐STAI‐6) is a six‐item, validated questionnaire measuring state anxiety [25]. Statements such as “I feel tense” and “I feel upset” were rated on a four-point Likert scale. Scores range from 20 to 80 with higher scores indicating greater anxiety. The normal score in the general population ranges between 34 and 36. A score >49 is considered clinically significant, although it does not necessarily represent a clinical diagnosis. Cronbach’s alpha was 0.89 (*n* = 560), indicating a high internal consistency.

### Illness Perceptions (BIPQ)

The eight-item BIPQ measured cognitive and emotional illness perceptions relating to HPV diagnosis [26]. Each item represented one illness perception dimension and was assessed on a rating scale from 0 to 10. The eight items included: consequences (*how much does your HPV affect your life?*), timeline (*how long do you think your HPV will last?*), personal control (*how much control do you feel you have over your HPV?*), treatment control (*how much do you think cervical screening can help your HPV?*), identity (*how much do you experience symptoms from your HPV?*), coherence (*how well do you feel you understand your HPV?*), emotional representation (*how much does your HPV affect you emotionally?*), and concern (*how concerned are you about your HPV?*). Higher scores indicated more negative beliefs about HPV for most dimensions, except for personal control and treatment control where higher scores represented more positive beliefs. The BIPQ has been shown to be a valid and reliable measure across a variety of health conditions [12].

### HPV-Related Symptom Attributions (Illness Perception Questionnaire-Revised subscale)

The Illness Perception Questionnaire-Revised (IPQ-R) symptom subscale was used to measure a more detailed list of HPV-related symptom attributions [9]. The IPQ-R symptom subscale asked participants whether they had experienced 15 general symptoms within the last 4 weeks, which they believed was related to their HPV (e.g. fatigue, nausea, and sore throat). Response options of “yes” or “no” were offered. Three additional symptoms were added to the illness identity scale from the IPQ-R: unusual bleeding, pain from sex, and vaginal discharge. These were listed as signs of cervical cancer in the routine result letters women received and might be considered plausible symptoms of HPV (which is in fact asymptomatic). The illness identity scale had good internal reliability as determined by the Kuder–Richardson 20 (KR-20) formula (=0.87), which is a special case of Cronbach’s alpha for dichotomous scores.

### Demographics

Demographic characteristics included self-reported education, ethnicity, and marital status. Age (years), index of multiple deprivation (IMD score and quintile; a multidimensional marker of area‐level deprivation), and NHS site were recorded from clinical records.

### Clinical Characteristics

Clinical characteristics included self-reported (nonvalidated) clinical diagnosis of a current anxiety disorder (yes, no, and prefer not to say) and current depression (yes, no, and prefer not to say). NHS data from clinical records provided information on test result (first HPV+/normal test result; or second or third consecutive HPV+/normal result at 12 month follow-up screen(s)).

### Statistical Analysis

Ten percent of data were checked independently by a member of the research team not involved in the initial data entry. Error rates were below the prespecified cutoff (<1% error for all outcomes), so no further action was taken. Statistical analyses were performed using SPSSv25 and MPlus v7.3 and a *p*-value <.05 was considered statistically significant. Demographic and clinical characteristics were descriptively assessed using one-way analysis of variance (ANOVA) and chi-squared tests. LPA [27] was used to assess whether underlying subgroups had similar or diverse illness perceptions about HPV. LPA is a model-ﬁtting method that uses probabilistic clustering and generates several model ﬁt statistics, providing a process for comparing the number of profiles. To select the most parsimonious number of profiles and maximize model ﬁt, a series of latent profile models were ﬁtted to the data. First, a single-profile model (based on the assumption that all participants had the same pattern of illness perceptions) was ﬁtted. This was followed by successive models, which increased by the unit of latent profiles until the results were no longer interpretable. To determine the best-fitting model, the interpretability of the model, sample size of each latent profile, and model fit statistics, including Akaike information criterion (AIC), Bayesian information criterion (BIC), adjusted BIC (ABIC), bootstrapped likelihood ratio test (BLR test), Lo–Mendell–Rubin likelihood ratio test (LMR LR test), and adjusted LMR LR test (ALMR LR test) were considered. Classification uncertainty was assessed by model entropy based on the posterior probabilities of latent profile membership. For the LMR LR test, ALMR LR test, and BLR test, a significant *p*-value indicated that the k profiles model fitted to the data better than the k−1 profile model. Once the best-fitting model was determined, women in our sample were assigned their most likely latent profile membership, which denoted their illness representation profile. Following latent profile membership assignment, multivariate analysis of variance (MANOVA) and post hoc tests were performed to examine differences in individual illness perceptions between latent profile groups.

Hierarchical multiple linear regression assessed the extent to which illness representations were associated with anxiety when controlling for relevant demographic and clinical characteristics. Demographic and clinical characteristics were entered in Model 1 as covariates. Illness representation profile was entered in Model 2 as the predictor variable to determine the extent to which each group contributed to the variance in anxiety, when controlling for demographic and clinical characteristics. These models included inverse-probability sample weight to account for measurement error from the determination of most likely latent class membership.

Data completeness was >95% for most outcomes and factors, except for anxiety (87%), IMD (94%), current depression (87%), current anxiety (87%), and the BIPQ items timeline (92%) and symptoms (93%). We used multiple imputation to account for missing data, and the model included psychological outcomes and all sociodemographic factors, which we assumed included all predictors of missingness. Demographic variables where participants had indicated “prefer not to say” or “other” were treated as missing in the multiple imputation model and main analyses. The final models were derived by fitting a regression model including all confounders, and estimates were combined using Rubin’s rules [28]. Sensitivity analysis compared complete data with multiple imputed data, with no substantive differences. Results are presented using imputed data.

### Patient and Public Involvement

Two patient and public involvement representatives reviewed the research protocol and all participant facing materials (information sheet, survey, and cover letter). They provided feedback on acceptability and structure of the content and design, which was integrated into final documents.

## Results

Of 2,702 women invited to take part, the majority were from NHS Greater Manchester (*n* = 2,090; 77.4%) as this was the largest recruitment site with geographical coverage across North England. The remainder (*n* = 612; 21.6%) were invited from NHS North West London, which was a smaller screening site but included a rich ethnic diversity in its catchment area. Overall, 646 women returned a questionnaire (*n* = 513 Manchester; *n* = 133 London) and were included in the analysis, yielding a response rate of 23.9% (24.5% Manchester; 21.7% London).

### Demographics and Sample Characteristics

Table 1 shows demographic and clinical characteristics. On average, women were 38.26 years (standard deviation [*SD*] = 11.86) and completed the survey 21.47 days (*SD* = 16.06) after receiving their result. They were predominantly White (British or other), had a current partner, and had received their first HPV+/normal test result. Educational attainment was roughly equally split between qualifications above and below degree level. Mean anxiety score was 47.18 (*SD* = 16.39), and 18.4% and 16.3% self-reported a current anxiety disorder and current depression diagnosis, respectively. Mean illness perception scores (out of 10) ranged from 1.31 (*SD* = 2.13) for personal control to 8.06 (*SD* = 2.55) for treatment control (see Table 3 for the full list). Relatively small proportions attributed specific symptoms to HPV (most were reported by <5%); however, the three most common were discharge (18.9%), unusual bleeding (12.4%), and pain during sex (10.4%; see Supplementary Table 1 for the full list).

**Table 1.** Demographic and clinical characteristics using imputed data (*N* = 646)

Demographics	
Age in years (*M*, *SD*)	38.26 (11.86)
Ethnicity (*N*, %)	
White (British or other)	526 (81.42)
Other ethnic group	96 (14.86)
Prefer not to say	24 (3.72)
Education (*N*, %)	
Qualification below degree	337 (52.17)
Degree level or higher	309 (47.83)
Marital status (*N*, %)	
Current partner	472 (73.07)
No current partner	174 (26.93)
IMD quintile (*N*, %)	
Quintile 1 (most deprived)	156 (25.70)
Quintile 2	176 (28.99)
Quintile 3	128 (21.09)
Quintile 4	88 (14.50)
Quintile 5 (least deprived)	59 (9.72)
NHS site (*N*, %)	
North West London	133 (20.59)
Greater Manchester	513 (79.41)
Current depression diagnosis (*N*, %)	
Yes	105 (16.25)
No	541 (83.75)
Current anxiety diagnosis (*N*, %)	
Yes	119 (18.42)
No	527 (81.58)
Test result (*N*, %)	
First HPV+/norm result	505 (78.17)
Second or third HPV+/norm result	141 (21.83)
Days to response (*M*, *SD*) *N* = 645	21.47 (16.06)

Education was dichotomized to represent below degree level (no formal qualification, O-Levels, CSE, ONC/BTEC, A-Level, higher education qualification) vs. degree level or higher (degree or higher degree). Ethnicity represented White (British or Other) vs. other (Black/African/Caribbean/Black British; Asian/Asian British; mixed/multiple ethnic groups; and other ethnic group) vs. Prefer not to say. Marital status was dichotomized as partner (married and cohabiting with a partner) vs. no partner (single, divorced, separated, and widowed). Test result was dichotomized as first vs. second or third consecutive HPV+/normal test result due to small numbers of women receiving a third consecutive test (*n* = 32).

*HPV* human papillomavirus; *IMD* index of multiple deprivation; *M* mean; *N* number of participants; *SD* standard deviation.

**Table 3.** Illness perceptions by latent profile (*N* = 646)

		Profile 1 (*n* = 248)	Profile 2 (*n* = 293)	Profile 3 (*n* = 105)		
	Whole sample (*n* = 646)	Adaptive representations	Negative representations	Negative somatic representations		
Illness perception	*M* (*SD*)	*M* (*SD*)			*F*	Posthoc tests
Consequences	4.06 (2.94)	1.71 (1.67)	5.30 (2.79)	5.67 (2.52)	206.03***	1 < 2,3
Timeline	5.96 (2.86)	4.59 (2.69)	6.74 (2.84)	7.72 (3.00)	69.40***	1 < 2, 3; 2 < 3
Personal control	1.31 (2.13)	1.57 (2.35)	1.13 (2.17)	0.94 (2.04)	4.51	–
Treatment control	8.06 (2.55)	8.21 (2.42)	7.99 (2.79)	8.35 (2.95)	1.06	–
Symptoms	1.45 (2.86)	0.43 (1.00)	0.71 (1.42)	5.62 (1.80)	782.01***	1, 2 < 3
Concern	7.05 (2.79)	4.64 (2.46)	8.56 (1.72)	8.38 (1.78)	303.45***	1 < 2, 3
Coherence	4.03 (3.01)	4.33 (2.98)	3.59 (3.19)	4.73 (3.27)	7.98***	1, 3 > 2
Emotion	5.40 (3.11)	2.50 (1.94)	7.25 (2.18)	5.87 (3.34)	312.02***	1 < 2, 3; 3 < 2

All illness perceptions are scored out of 10. A higher score indicates more serious perceived consequences, more chronic timeline beliefs, greater perceived personal and treatment control, more perceived symptoms, greater concern, a higher sense of coherence (understanding), and greater negative emotional response.

*M* mean; *SD* standard deviation, *F F*-test.

***p < .001.

### Identifying Illness Representations

LPA estimated model fit statistics for one to six profiles and allocated participants to each BIPQ profile. Three distinct groups of illness representations were identified. Whilst a four-profile solution had the best model fit as determined by the BIC and entropy values, the VLMR LR and ALMR LR tests were nonsignificant, suggesting that it did not improve on the three-profile model. Consequently, a three-profile model was selected since it was a significant improvement over a two-profile model (VLMR LR, ALMR LR, and BLRT all *p* < .001) and had reasonable sample sizes within each latent profile. See Table 2 for an overview of the model fit statistics.

**Table 2.** Model fit statistics for latent profile analysis of illness perceptions

Profile	AIC	BIC	ABIC	LLMR LR test *p*-value	ALMR LR test *p*-value	BLRT *p*-value	Entropy
1	24,253.19	24,324.72	24,273.92	–	–	–	–
2	23,355.52	23,467.29	23,387.92	<.001	<.001	<.001	0.83
3	22,903.32	23,055.33	22,947.38	<.001	<.001	<.001	0.87
4	22,684.11	22,876.35	22,739.83	.16	.16	<.001	0.84
5	22,589.33	22,821.81	22,656.72	.69	.70	<.001	0.86
6	22,439.51	22,712.29	22,518.57	.40	.40	<.001	0.87

*ABIC* sample size-adjusted BIC; *AIC* Akaike Information Criterion; *ALMR LR* adjusted Lo–Mendell–Rubin Likelihood Ratio Test; *BIC* Bayesian Information Criterion; *BLRT* Parametric Bootstrapped Likelihood Ratio Test For K-1 Profiles; *VLMR LR* Vuong–Lo–Mendell–Rubin Likelihood Ratio Test K-1 Profiles.

Estimated mean BIPQ scores (and 95% confidence intervals [CIs]) were generated from the LPA three-profile model (see Supplementary Table 2). Profile 1 (*n* = 248, 38.4%) was labeled “adaptive representations” as it was characterized by: almost no attributed symptoms to HPV; low consequences, personal control, and emotional response; and moderate coherence, concern, and chronic timeline. Profile 2 (*n* = 293, 45.4%) was labeled “negative representations” as participants displayed: almost no symptoms; low coherence and personal control; moderate consequences; and high chronic timeline, treatment control, concern, and emotional response. Profile 3 (*n* = 105, 16.2%) was labeled “negative somatic representations” as participants displayed similar representations to Profile 2, but with the notable addition of a moderate-high symptom score. Profile 1 (adaptive representations) was used as the reference group in the subsequent analyses as it was anticipated that this profile was most indicative of an adaptive response.

One-way MANOVA revealed significant differences in illness perceptions between the three profiles (observed means), *F*(16, 1270) = 157.78, *p* < .001; Wilk’s Ʌ = .11. Follow-up univariate ANOVA revealed that perceived consequences, timeline, symptoms, concern, coherence, and emotion differed significantly between the three latent profiles using Bonferroni adjustment to account for multiple tests (all *p* ≤ .006; 0.05/8 tests). Tukey post hoc tests showed that when compared with Profile 1 (adaptive representations), Profile 2 (negative representations) displayed significantly higher consequences (MD = 3.59, 95% CI = 3.12–4.06), higher timeline (MD = 2.15, CI = 1.60–2.71), higher concern (MD = 3.92, CI = 3.51–4.32), lower coherence (MD = −0.74, CI= −1.36 to −0.12), and higher emotional response (MD = 4.75, CI: 4.29–5.21). When compared with Profile 1, Profile 3 (negative somatic representations) showed higher consequences (MD = 3.96, CI = 3.36–4.56), higher timeline (MD = 3.13, CI = 2.42–3.83), higher symptoms (MD = 5.19, CI = 4.84–5.54), higher concern (MD = 3.74, CI = 3.22–4.25), and higher emotion (MD = 3.37, CI = 2.80–3.96). Profile 3 displayed higher symptoms (MD = 4.91, CI = 4.58–5.29), higher timeline (MD = 0.98, CI = 0.30–1.66), and lower emotion (MD = −1.38, CI = −0.81 to −1.94) when compared with Profile 2 (see Table 3).

Post hoc chi-square analyses were performed to compare proportions attributing specific symptoms to having HPV between the three latent profiles, and a *p*-value ≤.003 was considered significant to account for multiple tests using a Bonferroni adjustment (0.05/18 tests). Overall, the negative somatic illness representation profile (Profile 3) endorsed nearly all symptoms most frequently. As shown in Table 4, the most commonly endorsed were discharge (49.5%), unusual bleeding (32.4%), pain during sex (28.6%), pain (24.8%), fatigue (13.3%), and sleeping difficulties (11.4%).

**Table 4.** HPV-related symptom attributions by the latent profile (*N* = 646)

	Profile 1 (*n* = 248)	Profile 2 (*n* = 293)	Profile 3 (*n* = 105)	
	Adaptive representations	Negative representations	Negative somatic representations	Statistic
	*N* (%)			*X* ^2^
Discharge	21 (8.47)	49 (16.72)	52 (49.52)	82.81***
Unusual bleeding	12 (4.84)	34 (11.60)	34 (32.38)	51.87***
Pain during sex	12 (4.84)	25 (8.53)	30 (28.57)	46.65***
Pain (unspecified)	8 (3.23)	21 (7.17)	26 (24.76)	45.17***
Sleep difficulties	2 (0.81)	29 (9.90)	12 (11.43)	22.46***
Fatigue	2 (0.81)	22 (7.51)	14 (13.33)	23.47***
Loss of strength	0 (0)	13 (4.44)	8 (7.62)	16.01***
Upset stomach	3 (1.21)	10 (3.41)	8 (7.62)	9.68
Stiff joints	1 (0.40)	10 (3.41)	7 (6.67)	11.46**
Headaches	3 (1.21)	8 (2.73)	7 (6.67)	8.12
Nausea	0 (0)	10 (3.41)	6 (5.71)	11.92**
Dizziness	1 (0.40)	9 (3.07)	5 (4.76)	7.51
Weight gain	0 (0)	10 (3.41)	4 (3.81)	8.97
Sore throat	0 (0)	6 (2.05)	7 (6.67)	16.63***
Weight loss	0 (0)	6 (2.05)	4 (3.81)	7.90
Sore eyes	1 (0.40)	5 (1.71)	3 (2.86)	3.62
Breathlessness	0 (0)	5 (1.71)	3 (2.86)	5.88
Wheeziness	0 (0)	3 (1.02)	0 (0)	3.63

*N* number of participants; *X*^*2*^ = Pearson’s chi-square statistic.

**p < .003 to account for multiple tests; ***p < .001.

Supplementary Table 3 displays demographic and clinical characteristics for each of the three profiles. Only age, response time (days), education, and the proportions with a current anxiety disorder and current depression differed significantly across the three profiles (all *p* < .05).

### Associations Between Anxiety and Illness Representations

Univariate analyses revealed that women who had lower education, a current diagnosis of anxiety, a current diagnosis of depression, and had received their first HPV+/norm result reported significantly higher anxiety (all *p* < .05). There were also significant differences in anxiety between the three latent profiles (*F*(2, 642) = 100.87, *p* < .001). Post hoc tests using Bonferroni adjustment showed that when compared with Profile 1 (adaptive representations), Profile 2 (negative representations) and Profile 3 (negative somatic representations) had significantly higher anxiety scores (MD = 17.26, CI = 14.29–20.22, *p* < .001; and MD = 13.20, CI = 9.45–16.96, *p* < .001, respectively). Profile 2 showed significantly higher anxiety than Profile 3 (MD = 4.06, CI = 0.50–7.69, *p* < .05; see Table 5).

**Table 5.** Bivariate associations between descriptive characteristics and anxiety (*N* = 646)

	Anxiety	
		*r*
Age in years (*n* = 646)	–	−.03
IMD score (*n* = 646)	–	.07
Days to respond (*n* = 645)	–	−.38
	*M* (*SE*)	*F*
Education		
Degree or higher (*n* = 309)	45.69 (1.01)	5.11*
Below degree (*n* = 337)	48.54 (0.93)	
Ethnicity		
White (*n* = 526)	47.02 (0.73)	0.17
Other ethnic group (*n* = 96)	47.11 (0.67)	
Marital status		
Partner (*n* = 472)	46.20 (0.77)	1.11
No partner (*n* = 174)	48.29 (1.29)	
NHS site		
Manchester (*n* = 513)	47.18 (0.72)	0.07
London (*n* = 133)	47.16 (1.58)	
Test result		
First HPV+/normal result (*n* = 505)	47.89 (0.74)	4.48*
Second or third HPV+/normal result (*n* = 141)	44.62 (1.48)	
Current anxiety disorder		
Yes (*n* = 119)	51.91 (1.71)	14.87***
No (*n* = 526)	45.59 (0.73)	
Current depression		
Yes (*n* = 105)	51.85 (1.75)	12.59**
No (*n* = 541)	45.79 (0.72)	
Illness representation profiles		
Adaptive (*n* = 248)	36.48 (0.94)	100.87***
Negative (*n* = 293)	53.74 (0.87)	
Negative somatic (*n* = 105)	49.68 (1.30)	

*F F*-value; *HPV* human papillomavirus; *M* mean; *r* Pearson’s correlation; *SE* standard error.

*p < .05; **p < .01;***p < .001.

Table 6 displays the standardized regression coefficients (β), *R*, adjusted *R*^2^, and change in *R*^2^ after entering all variables into the hierarchal linear regression model. Model 1 included demographic and clinical variables and explained 3.7% of the variance (adjusted *R*^2^) in anxiety scores (*F*(9, 620) = 3.63, *p* < .001). Model 2 additionally included the three illness representation profiles and explained an additional 21.8% of the variance in anxiety, with the overall model explaining 25.6% of variance (*F*(11,620) = 20.36 *p* < .001). Both IMD score and test result were significantly associated with anxiety (β = −.09, *t* = −2.50, *p* < .05 and β = −.11, *t* = −2.81, *p* = <.01, respectively). Negative representations (Profile 2) and negative somatic representations (Profile 3) were also significantly associated with higher anxiety when compared with adaptive representations (Profile 1; β = .51, *t* = 12.44, *p* < .001 and β = .34, *t* = 8.27*, p* < .001, respectively). Overall, the final model indicated that greater deprivation, receiving a first HPV+/normal test result, and having negative or negative somatic representations were significantly associated with higher anxiety.

**Table 6.** Multiple hierarchical linear regression predicting anxiety (*N* = 646)

Parameter	Standardized coefficients		*R*	Adjusted *R*^2^	Change in *R*^2^
	Model 1	Model 2			
1.Demographics and clinical characteristics			.225	.037	
Age (years)	−.04	−.04			
IMD Score	.07	.09*			
Ethnicity	.03	<.001			
Education	.07	.03			
Marital status	−.03	−.02			
Test result	−.12**	−.11**			
NHS site	−.03	−.01			
Current anxiety disorder	.08	.10			
Current depression	.06	−.01			
2.Illness representations			.518	.256	.218***
Adaptive		ref			
Negative		.51***			
Negative somatic		.34***			

Demographic and characteristic variables coded as follows: ethnicity (1 = white; 2 = other ethnicity), education (1 = degree or higher; 2= below degree level), marital status (1 = no partner, 2 = partner), test result (1 = first result, 2= second or third result), and NHS site (1 = Greater Manchester, 2 = North West London).

*IMD* index of multiple deprivation; *R* = correlation coefficient.

*.05, **.01, ***.001.

## Discussion

To the best of our knowledge, this is the first study to identify illness representation profiles following an HPV+/normal cytology cervical screening result and to explore their associations with anxiety. The study demonstrated that different groups of women testing HPV+/normal have different illness representations profiles, consistent with the theoretical construct originally proposed by Leventhal et al. [8, 16]. In our sample, three distinct profiles of illness representations were identified (termed “adaptive,” “negative,” and “negative somatic”), which differed significantly in their patterns of illness perceptions. We also found that these illness representation profiles accounted for 21.8% of the variance in anxiety, after adjusting for relevant demographic and clinical characteristics. When compared with adaptive representations, women with negative representations and negative somatic representations reported statistically significantly higher anxiety, with clinically significant between-group differences (scores of >49 on the S-STAI-6 for negative [somatic] representations). Our identified latent profiles of illness representations may provide insights for targeting maladaptive beliefs in interventions or patient communications, potentially helping to reduce anxiety within subgroups of highly anxious women following an HPV+/normal result.

Women with adaptive representations accounted for around 38% of our sample and had the lowest mean anxiety score within the normal population range (mean S-STAI-6 of 36.5; normal range 36–38) [4]. Notably, this group was characterized by low perceived consequences of HPV, which suggests that cervical cancer or sexual concerns were unlikely. Despite a mean score of 4.3 out of 10 for coherence (perceived understanding of HPV), their wider illness representation profile appeared to reflect a relatively accurate interpretation of their result. They perceived a moderate timeline, consistent with the fact that HPV usually clears within 1–2 years; almost no symptoms, which is consistent with HPV being asymptomatic; and low-moderate concern when relative risk of cervical cancer is higher-than-average but absolute risk is very low. Overall, this pattern of illness perceptions may, therefore, characterize an adaptive response. However, we were unable to rule out the possibility that “adaptive” representations could be associated with negative behavioral impacts. If some women were in fact apathetic or avoidant, they may be less likely to reattend for 12 month follow-up screening, which would reduce the mortality-reduction benefit of HPV primary screening. Emotional detachment has been associated with favorable cognitive-affective outcomes by initially reducing distress in patients with chronic illness and disability [29, 30] but could also potentially lead to unhelpful behaviors through disengagement [31–33].

Women with negative representations accounted for the greatest proportion of the sample (around 45%) and, in contrast with the adaptive representations group, were characterized by higher perceived consequences, concern, and emotional response, as well as lower coherence. Consistent with the wider HPV literature, these women appeared focused on the timeline of HPV and its potential consequences and reported high emotional impact and concern [34–37]. Unsurprisingly, when compared with the adaptive representation group, they had markedly higher anxiety (mean difference of 17.3), as well as descriptively clinically significant anxiety (mean of 53.7; >49). Even after adjusting for demographic and clinical characteristics, including a self-reported current anxiety disorder, this profile showed the strongest association with anxiety (based on beta-weight). Some systematic reviews of illness representations across other conditions suggest that perceived consequences and emotion are among the most important drivers of health-related outcomes [14, 24]. Our findings appear to support the central role of illness representations in highly anxious women following HPV diagnosis and the CSM as a useful cognitive-affective framework.

Lastly, women with negative somatic representations (16%) presented with very similar illness perceptions as negative representations but with notably higher perceived HPV-related symptoms. These women also had higher anxiety than the adaptive representations group, which was descriptively clinically significant (mean anxiety score of 49.7; >49). HPV is an asymptomatic infection and the only evidenced reason for symptoms is early stage cervical cancer (e.g., abnormal vaginal bleeding or discharge). Given that women in this study had a normal cytology result, cancer is extremely unlikely unless it was a false negative, which is rare. HPV-related symptom perceptions are, therefore, almost certainly due to misattributions. On descriptive examination of individual symptoms reported, the three most commonly endorsed were discharge (50%), unusual bleeding (32%), and pain during sex (29%). These three items were added to the BIPQ to assess symptoms that might plausibly be attributed to HPV and are listed as possible symptoms of cervical cancer (for which help should be sought) in the routine result letters women received at screening. This suggests that, for most, misattributions of conceivable HPV-related symptoms were more likely than affect-induced common everyday symptoms, such as fatigue.

Overall, our findings identify areas that can be used by health care professionals and policymakers to improve communication of HPV+/normal results and help alleviate the concerns of the most highly anxious women. Targeted messages in patient communications (e.g., result letters or leaflets) could aim to address negative or negative somatic illness representations by providing supportive information on potential consequences of HPV (e.g., cancer risk and sexual relationships) and emphasizing that HPV is asymptomatic. Online HPV-focused self-help interventions or brief psycho-education interventions could be developed to improve coherence (understanding of HPV) and emotional representations. Cognitive behavioral treatment approaches could, for example, be integrated into these interventions and methodologically strengthened through the use of formal development methods like Intervention Mapping [38]. Brief interventions could be made available through health care or third sector websites and signposted to in routine result letters.

### Limitations

Inherent limitations associated with our cross-sectional design may be particularly problematic when interpreting dynamic illness representations, which can evolve over time [39, 40] and over the trajectory of a condition [41, 42]. We likely captured acute psychological responses as supported by our 21 day average time from result to survey completion; however, some women responded up to 111 days later, introducing the possibility of illness representations evolving to a more chronic state. Furthermore, almost a fifth of our sample had tested HPV+/normal for the second or third consecutive time, which, although adjusted for in analyses, may have incorporated chronic or cyclical illness representations. Some women may have visited a GP or attended colposcopy (colposcopy is recommended after a third consecutive result) before completing our survey, which could have influenced their illness representations if they were given additional information [43]. Our low response rate (24%) may limit inferences to the wider HPV+/normal population, especially given that nearly half of our sample were educated to at least degree level. Findings may not be generalizable to harder-to-reach populations (such as those without access to universal health care or fixed residence) given that women had to be registered with a GP to take part in cervical screening and then mailed a survey to a registered address. Lastly, the mean anxiety score was also higher than previous research using similar recruitment methods in the same population (*M* = 47.2 vs. *M* = 38.3, respectively) [4]. This likely reflects higher sensitivity to result exposure in our study due to faster time to approach but could represent an opt-in bias, with the most anxious women taking part.

## Conclusion

Three distinct illness representation profiles were identified in women testing HPV-positive with normal cytology at routine cervical cancer screening, explaining 21.8% of the variance in anxiety after controlling for demographic and clinical characteristics. These illness representation profiles corresponded to women displaying both normal and clinically significant anxiety levels, with between-group differences suggesting that perceived consequences, timeline, concern, emotion, and symptoms related to HPV may be important drivers of anxiety. Characteristics specific to subgroups of highly anxious women (i.e., Profiles 2 and 3) could be used to target information in routine patient communications (e.g., test result letters and leaflets). Future research should use longitudinal designs to understand the trajectory of illness representations from HPV diagnosis through to clearance versus persistence whilst incorporating other relevant affective and behavioral outcomes to assess a more holistic picture.

## Supplementary Material

kaab022_suppl_Supplementary_Table_1

kaab022_suppl_Supplementary_Table_2

kaab022_suppl_Supplementary_Table_3
