# Supplementary material for: Distinct Illness Representation Profiles Are Associated With Anxiety in Women Testing Positive for Human Papillomavirus
Source: Ann Behav Med. 2021 Apr 21;56(1):78–88. doi: 10.1093/abm/kaab022 (PMC8691260; doi:10.1093/abm/kaab022)
Supplement: kaab022_suppl_Supplementary_Table_1 [file kaab022_suppl_supplementary_table_1.docx]

Supplementary Table 1 - List of individual HPV-related symptom attributions.

| **HPV-related symptom attributions (N, %)** | | |
| --- | --- | --- |
|  | Discharge | 122 (18.89) |
|  | Unusual bleeding | 80 (12.38) |
|  | Pain during sex | 67 (10.37) |
|  | Pain (unspecified) | 55 (8.51) |
|  | Sleeping difficulties | 43 (6.66) |
|  | Fatigue | 38 (5.88) |
|  | Loss of strength | 21 (3.25) |
|  | Upset stomach | 21 (3.30) |
|  | Stiff joints | 18 (2.79) |
|  | Headaches | 18 (2.79) |
|  | Nausea | 16 (2.48) |
|  | Dizziness | 15 (2.32) |
|  | Weight gain | 14 (2.17) |
|  | Sore throat | 13 (2.01) |
|  | Weight loss | 10 (1.55) |
|  | Sore eyes | 9 (1.39) |
|  | Breathlessness | 8 (1.24) |
|  | Wheeziness | 3 (0.46) |

The statistics represent the number (N) and percentage (%) of the sample who endorsed that they experienced a symptom and then endorsed ‘yes’ they believed this symptom was related to their HPV.
