# Supplementary material for: Distinct Illness Representation Profiles Are Associated With Anxiety in Women Testing Positive for Human Papillomavirus
Source: Ann Behav Med. 2021 Apr 21;56(1):78–88. doi: 10.1093/abm/kaab022 (PMC8691260; doi:10.1093/abm/kaab022)
Supplement: kaab022_suppl_Supplementary_Table_2 [file kaab022_suppl_supplementary_table_2.docx]

Supplementary Table 2 – Estimated means (95% confidence intervals) of illness perceptions for the 3-profile LPA solution (N=646).

| **.** | **Profile-1 (n=248) .** | **Profile-2 (n=293) .** | **Profile-3 (n=105) .** |
| --- | --- | --- | --- |
|  | **Positive Representations .** | **Negative Representations .** | **Negative Somatic Representations .** |
| **Illness Perception** | M (95% CI) | | |
| Consequences | 1.72 (1.47-1.96) | 5.43 (4.89-5.98) | 5.93 (5.34-6.52) |
| Timeline | 4.85 (4.10-4.92) | 6.81 (6.44-7.19) | 7.09 (6.51-7.68) |
| Personal control | 1.57 (1.26-1.88) | 1.08 (0.83-1.34) | 1.18 (0.82-1.53) |
| Treatment control | 8.17 (7.87-8.48) | 8.09 (7.77-8.42) | 7.75 (7.20-8.29) |
| Symptoms | 0.44 (0.28-0.59) | 0.49 (0.36-0.61) | 6.11 (5.79-6.43) |
| Concern | 4.53 (4.00-5.07) | 8.73 (8.47-9.00) | 8.55 (8.14-8.96) |
| Coherence | 4.34 (3.94-4.75) | 3.64 (3.26-4.01) | 4.28 (3.66-4.90) |
| Emotion | 2.48 (2.06-2.89) | 7.37 (6.93-7.80) | 7.07 (6.57-7.57) |

*Note. M* = estimated mean, *CI* = 95% confidence interval, *N* = number of participants.

All illness perceptions are scored out of 10.
