# Supplementary material for: Distinct Illness Representation Profiles Are Associated With Anxiety in Women Testing Positive for Human Papillomavirus
Source: Ann Behav Med. 2021 Apr 21;56(1):78–88. doi: 10.1093/abm/kaab022 (PMC8691260; doi:10.1093/abm/kaab022)
Supplement: kaab022_suppl_Supplementary_Table_3 [file kaab022_suppl_supplementary_table_3.docx]

Supplementary Table 3 – Descriptive and clinical characteristics by latent profile (N=646).

|  | **Profile-1 (n=248)** | | **Profile-2 (n=293)** | | **Profile-3 (n=105)** |  |
| --- | --- | --- | --- | --- | --- | --- |
|  | **Positive Representations** | | **Negative Representations** | | **Negative Somatic Representations** |  |
|  | **M (SD)** | | | | | ***F*** |
| **Age** | 38.48 (13.06) | | 38.25 (12.16) | | 41.31 (13.74) | 3.03* |
| **IMD Score** | 26.14 (15.06) | | 25.20 (17.94) | | 23.90 (19.03) | 0.81 |
| **Days to Respond to survey (N=644)** | 22.17 (17.08) | | 19.44 (14.88) | | 25.07 (18.97) | 6.05** |
|  | **N (%)** | | | | | ***X²*** |
| **Education** |  | | | | |  |
| Degree or higher | 132 (53.23) | | 141 (48.12) | | 36 (34.29) | 10.40** |
| Below degree | 116 (46.77) | | 152 (51.88) | | 69 (65.71) |  |
| **Ethnicity** |  | | | | |  |
| White | 206 (86.19) | | 237 (84.34) | | 83 (81.37) | 1.29 |
| Other ethnic group | 33 (13.81) | | 44 (15.76) | | 19 (18.63) |  |
| **Marital Status** |  | | | | |  |
| No Partner | 62 (25.00) | | 79 (26.96) | | 34 (32.38) | 1.92 |
| Partner | 186 (75.00) | | 214 (73.04) | | 71 (68.62) |  |
| **NHS Site** |  | | | | |  |
| Manchester | 197 (79.44) | | 237 (80.89) | | 79 (75.24) | 1.51 |
| London | 51 (20.56) | | 56 (19.11) | | 26 (24.76) |  |
| **Test Result** |  | | | | |  |
| 1^st^ result | 185 (74.60) | | 237 (80.89) | | 83 (79.05) | 5.42 |
| 2^nd^ or 3^rd^ result | 63 (25.40) | | 56 (19.11) | | 22 (20.95) |  |
| **Current Anxiety Disorder** | |  | |  | |  |
| Yes | 35 (14.11) | | 53 (18.09) | | 32 (30.48) | 13.71** |
| No | 213 (85.89) | | 240 (81.91) | | 73 (69.52) |  |
| **Current Depression** |  | |  | |  |  |
| Yes | 25 (10.08) | | 50 (17.06) | | 30 (28.57) | 18.63*** |
| No | 223 (89.92) | | 243 (83.94) | | 75 (71.43) |  |

M= mean; SD= standard deviation; N = number of participants; % = percentage; *F*= F-value; *X²*= chi-squared value.

[*](javascript:;)*p* < 0.05; [**](javascript:;)*p* < 0.01;****p*<.001.
